# Response to: Comment on “Clinical and Pathological Characteristics of Autoimmune Hepatitis with Acute Presentation”

**DOI:** 10.1155/2021/9573837

**Published:** 2021-10-13

**Authors:** Yi Shen, Li Yang

**Affiliations:** Department of Gastroenterology & Hepatology, West China Hospital, Sichuan University, Chengdu, Sichuan 610041, China

With great interest, we read the letter by Muratori [1], commenting on our study “Clinical and Pathological Characteristics of Autoimmune Hepatitis with Acute Presentation.” [2] The author reports on his group's experience of the differences between acute and chronic autoimmune hepatitis (AIH) [3] and concludes that acute AIH patients did not have higher IgG level when compared to chronic patients. They also report that acute patients in their study had a better prognosis after immunosuppressive treatment.

We are glad to see that the Italian researchers also accepted the criterion of acute-presentation AIH, which makes it possible to compare our two different national studies. As for the different results of IgG levels between our two studies, we had suggested a possible reason for this in the “Discussion” part of our article to provide clarity. In our study, we focused on the differences between the acute-presentation and the chronic-presentation AIH; the patients with acute-on-chronic AIH were also included in the acute-presentation group, which explained the result of IgG in our study, even though IgG is not an acute-phase protein. In addition, we undertook a subgroup analysis to compare the fibrosis stages between the two groups; the result here also showed no significant difference between the acute-presentation and the chronic-presentation group. As for the accuracy of the diagnosis, we think the value of liver pathological judgement for AIH patients is more important than the existence of smooth muscle antibody. In our department, almost every suspected AIH patient underwent one or two liver biopsies in order to determine the diagnosis, and because of the relative low test rate of the smooth muscle antibody, we include these data in the final article. The prognosis of AIH is also a major concern for our department, and the follow-up of all AIH patients after treatment is therefore ongoing. The therapeutic response and the recurrence of AIH are important issues which require further exploration.

In conclusion, the best criterion for the discrimination of acute AIH and chronic AIH is still a controversial problem, and the differences between the two states of AIH still attract our curiosity. We anticipate that we will be able to share these results with the Italian researchers in the future.
